# Supplementary material for: Lamellar Polypyrene Based on Attapulgite–Sulfur Composite for Lithium–Sulfur Battery
Source: Membranes (Basel). 2021 Jun 29;11(7):483. doi: 10.3390/membranes11070483 (PMC8306344; doi:10.3390/membranes11070483)
Supplement: Supplementary file 1 [file membranes-11-00483-s001.zip › membranes-1257870-supplementary.pdf]

membranes

MDPI

Supplementary Material

Lamellar Polypyrene Based on Attapulgite–Sulfur Composite for Lithium-Sulfur Battery

Jing Wang [1,*], Riwei Xu [1], Chengzhong Wang [1], Jinping Xiong [1,2,*]

[1] Beijing Key Laboratory of Electrochemical Process and Technology for Materials, Beijing University of Chemical Technology, Beijing 100029, China; xurw@mail.buct.edu.cn (R.X.); czwang@mail.buct.edu.cn (C.W.)

[2] Fujian Provincial Key Laboratory of Ecological Industry Green Technology, Wuyi University, Wuyishan 354399, China

* Correspondence: 2020700016@mail.buct.edu.cn (J.W.); xiongjp@mail.buct.edu.cn (J.X.)

Table S1. The comparison of the related work.

Related work	Synthetic method	Mixing with Sulfur	Nano structure	Electrochemical application
PPy-AT [1]	Chemical	No	Nanotube	No
Bentonite/S [2]	Physical	Grinding and co-heating	Irregular sheet structure	0.2 C, initial discharge specific capacity: 796 mAh; after 100 cycles: 489mAh.
ATTP@GNs/S [3]	Physical+Chemical	Grinding and co-heating	Nanosheets	0.1 C, initial discharge specific capacity: 1143.9 mAh/g; after 100 cycles: 512.0 mAh/g
AT-S-PPy	Physical+Chemical	Absorption and co-heating	Lamellar-like structure	0.1C, initial discharge specific capacity: 1175 mAh/g; after 100 cycles: 518 mAh/g.

References

1. Zhou, W.; Yu, Y.; Chen, H.; Disalvo, F.J.; Abruña, H.D. Yolk–Shell Structure of Polyaniline-Coated Sulfur for Lithium–Sulfur Batteries. *J. Am. Chem. Soc.* **2013**, *135*, 16736–16743. https://doi:10.1021/ja409508q.
2. Simmonds, A.G.; Griebel, J.J.; Park, J.; Kim, K.R.; Chung, W.J.; Oleshko, V.P.; Kim, J.; Kim, E.T.; Glass, R.S.; Soles, C.L.; et al. Inverse Vulcanization of Elemental Sulfur to Prepare Polymeric Electrode Materials for Li–S Batteries. *ACS Macro Lett.* **2014**, *3*, 229–232. https://doi:10.1021/mz400649w.
3. Wang, J.L.; Yang, J.; Xie, J.Y. A novel conductive polymer-sulfur composite cathode material for rechargeable lithium batteries. *Adv Mater*. **2002**, *14*, 963–965. https://doi: 10.1002/1521-4095(20020705)14:13/14<963::AID-ADMA963>3.0.CO;2-P.
